# Improving performance of the Tariff Method for assigning causes of death to verbal autopsies

**DOI:** 10.1186/s12916-015-0527-9

**Published:** 2015-12-08

**Authors:** Peter Serina, Ian Riley, Andrea Stewart, Spencer L. James, Abraham D. Flaxman, Rafael Lozano, Bernardo Hernandez, Meghan D. Mooney, Richard Luning, Robert Black, Ramesh Ahuja, Nurul Alam, Sayed Saidul Alam, Said Mohammed Ali, Charles Atkinson, Abdulla H. Baqui, Hafizur R. Chowdhury, Lalit Dandona, Rakhi Dandona, Emily Dantzer, Gary L. Darmstadt, Vinita Das, Usha Dhingra, Arup Dutta, Wafaie Fawzi, Michael Freeman, Sara Gomez, Hebe N. Gouda, Rohina Joshi, Henry D. Kalter, Aarti Kumar, Vishwajeet Kumar, Marilla Lucero, Seri Maraga, Saurabh Mehta, Bruce Neal, Summer Lockett Ohno, David Phillips, Kelsey Pierce, Rajendra Prasad, Devarsatee Praveen, Zul Premji, Dolores Ramirez-Villalobos, Patricia Rarau, Hazel Remolador, Minerva Romero, Mwanaidi Said, Diozele Sanvictores, Sunil Sazawal, Peter K. Streatfield, Veronica Tallo, Alireza Vadhatpour, Miriam Vano, Christopher J. L. Murray, Alan D. Lopez

**Affiliations:** Institute for Health Metrics and Evaluation, University of Washington, 2301 Fifth Avenue, Suite 600, Seattle, WA 98121 USA; University of Queensland, School of Population Health, Level 2 Public Health Building School of Population Health, Herston Road, Herston, QLD 4006 Australia; National Institute of Public Health, Universidad 1299 Buena Vista, 62115 Cuernavaca, Morelos Mexico; Institute for International Programs, Johns Hopkins University, Bloomberg School of Public Health, 615 N Wolfe Street, Baltimore, MD 21205 USA; Community Empowerment Lab, Shivgarh, India; The INCLEN Trust International, New Delhi, India; International Center for Diarrhoeal Disease Research, Dhaka, Bangladesh; Public Health Laboratory Ivo de Carneri (PHL-IdC), PO Box 122, Wawi Chake Chake Pemba, Zanzibar, Tanzania; Public Health Foundation of India, Plot 47, Sector 44, Gurgaon, 12002 National Capital Region India; Malaria Consortium Cambodia, 113 Mao Tse Toung, Phnom Penh, Cambodia; Department of Pediatrics, Stanford University School of Medicine, Stanford, CA 94304 USA; CSM Medical University, Shah Mina Road, Chowk Lucknow, Uttar Pradesh 226003 India; Harvard School of Public Health, 677 Huntington Avenue, Boston, MA 02115-6018 USA; Ipas, Chapel Hill, NC 27515 USA; Papua New Guinea Institute of Medical Research, Goroka, Papua New Guinea; The George Institute of Global Health, University of Sydney, Sydney, NSW 2000 Australia; Research Institute for Tropical Medicine, Corporate Avenue, Muntinlupa City, 1781 Philippines; Cornell University, Division of Nutritional Sciences, 314 Savage Hall, Ithaca, NY 14853 USA; Muhimbili University of Health and Allied Sciences, United Nations Road, Dar es Salaam, Tanzania; University of Melbourne, School of Population and Global Health, Building 379, 207 Bouverie Street, Parkville, VIC 3010 Australia; Royal Prince Albert Hospital, Sydney, Australia; Imperial College, London, UK; George Institute of Global Health India, Hyderabad, India

**Keywords:** Verbal autopsy questionnaire, Mortality surveillance, Causes of death

## Abstract

**Background:**

Reliable data on the distribution of causes of death (COD) in a population are fundamental to good public health practice. In the absence of comprehensive medical certification of deaths, the only feasible way to collect essential mortality data is verbal autopsy (VA). The Tariff Method was developed by the Population Health Metrics Research Consortium (PHMRC) to ascertain COD from VA information. Given its potential for improving information about COD, there is interest in refining the method. We describe the further development of the Tariff Method.

**Methods:**

This study uses data from the PHMRC and the National Health and Medical Research Council (NHMRC) of Australia studies. Gold standard clinical diagnostic criteria for hospital deaths were specified for a target cause list. VAs were collected from families using the PHMRC verbal autopsy instrument including health care experience (HCE). The original Tariff Method (Tariff 1.0) was trained using the validated PHMRC database for which VAs had been collected for deaths with hospital records fulfilling the gold standard criteria (validated VAs). In this study, the performance of Tariff 1.0 was tested using VAs from household surveys (community VAs) collected for the PHMRC and NHMRC studies. We then corrected the model to account for the previous observed biases of the model, and Tariff 2.0 was developed. The performance of Tariff 2.0 was measured at individual and population levels using the validated PHMRC database.

**Results:**

For median chance-corrected concordance (CCC) and mean cause-specific mortality fraction (CSMF) accuracy, and for each of three modules with and without HCE, Tariff 2.0 performs significantly better than the Tariff 1.0, especially in children and neonates. Improvement in CSMF accuracy with HCE was 2.5 %, 7.4 %, and 14.9 % for adults, children, and neonates, respectively, and for median CCC with HCE it was 6.0 %, 13.5 %, and 21.2 %, respectively. Similar levels of improvement are seen in analyses without HCE.

**Conclusions:**

Tariff 2.0 addresses the main shortcomings of the application of the Tariff Method to analyze data from VAs in community settings. It provides an estimation of COD from VAs with better performance at the individual and population level than the previous version of this method, and it is publicly available for use.

**Electronic supplementary material:**

The online version of this article (doi:10.1186/s12916-015-0527-9) contains supplementary material, which is available to authorized users.

## Background

Reliable data on the distribution of causes of death (COD) in a population are fundamental to good public health practice [1]. Ideally, COD data are based on accurate medical certification and registration of all deaths [2]. However, many, if not most, resource-poor countries lack adequate systems for the collection, tabulation, and dissemination of vital statistics on causes of death in their populations [3]. In the absence of comprehensive medical certification of deaths, the only feasible way to collect essential mortality data is verbal autopsy (VA), whereby relatives of the deceased respond to a questionnaire about the medical history of the decedent and of the terminal illness (the illness that led directly to death).

Methods for assigning the COD to VAs can be separated into two broad groups: those based on expert judgment of physicians and empirical methods that are data-driven. The first group includes physician-coded VAs (PCVA) [4] and InterVA, a computer program based on expert judgment [5]. The second group uses a data-driven approach, exploring patterns of responses on actual answers to verbal autopsies to ascertain the cause of death. This group includes methods such as King–Lu [6], Tariff Method [7], and Random Forest [8]. The last two were developed as part of the Population Health Metrics Research Consortium (PHMRC) gold standard verbal autopsy validation study [9].

With most analytic methods it is not possible to scrutinize the relationships between responses to individual items in the VA questionnaire and the different causes of death systematically. Tariff Method, on the other hand, is a simple additive algorithm based on a score, or tariff, for each question item-COD pair that performs as well or better than other analytic methods when validated against “gold standard” deaths for which the cause has been reliably established [7].

The PHMRC study [9] selected hospital deaths that met gold standard clinical criteria and compared diagnoses from the decedents’ medical records with VAs obtained from the families of the deceased. Necessarily, all decedents in the PHMRC database had had contact with health services that had the appropriate facilities and were otherwise capable of making reliable diagnoses. The PHMRC study assumes that the attributes of specific diseases leading to death in a hospital are sufficiently similar to the attributes of the same diseases leading to deaths in the community in order to draw conclusions about causes of death in the community. The principal potential application of VA is in community or population studies, where decedents can be expected to have had a range of experiences with health services. It is possible that exposure to the health care system may have influenced either the course of the illness itself or else affected responses to items in the questionnaire. The Tariff Method [7] addressed this limitation by classifying responses to questions in the PHMRC verbal autopsy instrument (VAI) according to whether they did or did not depend on the contact that relatives of the deceased person may have had with health services, namely the health care experience (HCE), so that performance could be reported as being with or without HCE.

Tariff Method was included in a recently published study of the comparative performance of six different methods for assigning COD to VAs [10]. Although the performance of the method in this comparative study was far superior to other diagnostic procedures commonly used, questions have been raised about the external validity of empirical methods developed from the PHMRC database [11]. Here we describe in detail the development of the updated Tariff Method we refer to as Tariff 2.0 and address issues of external validity. In our view, the processes of development and validation of empirical methods have been poorly understood by certain commentators and it is important that this be corrected if the full potential of well-performing automated VA diagnostic methods for reducing ignorance about causes of death is to be realized.

Steps in the development of Tariff 2.0 were: 1) testing Tariff 1.0 by using it to assign CODs to VAs collected in household surveys (community VAs); 2) revision and retraining of Tariff 1.0 using validated VAs (VAs that had been collected for deaths with hospital records fulfilling the gold standard criteria from the PHMRC gold standard database); 3) retesting Tariff 1.0 using community VAs and further revising the Tariff 1.0 to create Tariff 2.0; and 4) assessing the performance of Tariff 2.0 using the validation database at individual and population levels using as metrics chance-corrected concordance (CCC) and cause-specific mortality fraction (CSMF) accuracy.

## Methods

### PHMRC gold standard validation study database

The general methodology of the PHMRC study has been described in detail elsewhere [9] and is summarized here for convenience. VAs were collected from six sites in four countries: Andhra Pradesh and Uttar Pradesh in India; Bohol in the Philippines; Mexico City in Mexico; and Dar es Salaam and Pemba Island in Tanzania. Gold standard clinical diagnostic criteria for hospital deaths were specified for a target cause list of 53 adult, 27 child, and 13 neonatal causes, including stillbirths. Deaths with hospital records fulfilling the gold standard criteria were identified in each of the sites. Families were then interviewed about the events leading to each of these deaths using the PHMRC VAI [9]. Interviewers were blinded to the COD assigned in the hospital. The PHMRC database contains 12,501 verbal autopsies with gold standard diagnoses (7,846 adults, 2,064 children, 1,586 neonates, and 1,005 stillbirths). All data collection procedures were approved by the Internal Review Board of the University of Washington, Seattle, WA, USA; School of Public Health, University of Queensland; George Institute for Global Health, Hyderabad, India;  National Institute  of Public Health, Mexico;  Research Institute for Tropical Medicine, Alabang, Metro Manila, Philippines; Muhimbili University, Tanzania; Public Health Laboratory Ivo de Carneri, Tanzania; and  CSM Medical University, India . All information on VAs was collected after obtaining signed consent from the informants.

The target cause list was developed from World Health Organization (WHO) estimates of the leading CODs in developing countries in 2004 [12]. COD categories were based on the International Classification of Diseases (ICD) and are mutually exclusive and collectively exhaustive. The original cause list for the validation study was 53 for adults, 27 for children, and 13 for neonates (plus stillbirths). The number of causes in the target list was reduced; firstly, because there were insufficient cases for certain causes and secondly, because analytic methods were unable to discriminate between causes. The first reduction created an analysis cause list, which was used to test diagnostic algorithms, and the second, a reporting cause list containing 34 adult, 21 child, and 11 neonatal causes (including stillbirths) for output from the Tariff 1.0 [9]. The number of neonatal causes was further reduced from 11 to 6 for the updated version of Tariff [10] because of the use of combinations of causes that did not map to the ICD. In the further development of Tariff 2.0 it was realized that neonatal deaths with sepsis had been wrongly recoded in the reduction from 11 to 6 causes. The result has been to change the number of neonatal deaths by COD. Because prenatal deliveries with both sepsis and birth asphyxia could not be recoded to a list with single COD, 34 deaths were dropped from the test/training analyses. The COD lists are shown in Additional file 1. Reductions of the cause list preceded any development of the item-reduced instrument.

Changes to the categorization of neonatal causes and the further accumulation of community deaths has meant that there are differences in the detail of performance metrics between this paper and the comparison of methods for cause assignment published in 2014 for neonates. None of these changes is substantial, however, and none affects the conclusion we draw from this analysis.

The PHMRC VAI includes both closed-ended questions and an open-ended narrative. Question items were based on the closed-ended questions and cover: 1) symptoms of the terminal illness; 2) diagnoses of chronic illnesses obtained from health service providers (as reported by respondent as communicated to them by the health service provider, not obtained through record linkage); 3) risk behaviors (tobacco and alcohol); and 4) details of any interactions with health services. Text items were based on open-ended narrative using a text mining procedure that identifies key words and groups words with the same or similar meanings to create them. Performance was reported as being 1) with HCE and 2) without HCE, respectively. The former was based on analysis of all question and text items, whereas the latter was based on an analysis of question items on symptoms and risk behaviors only. Table 1 classifies items by whether responses were treated as being dependent or not on HCE.

**Table 1:** Classification of questionnaire items according to dependency on health care experience (HCE)

Type of item	Source	HCE dependent	Not HCE dependent
Question items	Closed-ended questions	History of chronic illness	Symptoms
		Interaction with health services	Risk behaviors
Text items	Open-ended narrative	Text	

### Community VA data

The development of Tariff 1.0 had been based on the PHMRC validation database and thus all deaths had occurred in hospital. Our initial aim in developing Tariff 2.0 was to review the cause distributions of deaths in community VAs using Tariff 1.0 and to see whether these distributions were plausible. This review was based on the examination of 12,528 VAs, not linked to gold standard hospital data, collected from community samples using the PHMRC VAI. VAs of 3,067 deaths, occurring within 5 years of interview, were collected from household surveys in Mexico City in Mexico, Andhra Pradesh in India, Pemba in Tanzania, and Bohol in the Philippines, as part of the PHMRC study [13]. A further 9,461 VAs were collected in Chandpur and Comilla Districts in Bangladesh, in Central and Eastern Highlands Provinces in Papua New Guinea, and in Bohol Province in the Philippines, as part of a study funded by the National Health and Medical Research Council (NMHRC) of Australia. The age-site distribution of these deaths is shown in Table 2. The performance of Tariff 2.0 could only be compared with that of Tariff 1.0 by using the PHMRC gold standard database.

**Table 2:** Number of community verbal autopsies without a gold standard cause of death by site and module used to test the Tariff Method

Grant	Site	Adult	Child	Neonate	Total
PHMRC	Andhra Pradesh, India	426	14	21	461
	Bohol, Philippines	847	34	21	902
	Mexico City, Mexico	1,104	51	43	1,198
	Pemba, Tanzania	303	123	80	506
	*Subtotal*	*2,680*	*222*	*165*	*3,067*
NHMRC	Chandpur, Bangladesh	3,440	242	355	4,037
	Bohol, Philippines	4,295	205	196	4,696
	Papua New Guinea	572	100	56	728
	*Subtotal*	*8,307*	*547*	*607*	*9,461*
	Total	10,987	769	772	12,528

### Tariff method

The premise of the Tariff Method is that individual question and text items are consistently associated with particular causes of death. In the Tariff Method, the association between each item-cause pair is quantified. The first step in quantification is to develop a matrix of endorsement rates for item-cause pairs based on the analysis cause list. An item in the VAI is said to have been endorsed if the response was “yes”. The tariff itself reflects the relationship between the endorsement rate for a particular item (*j*) and a particular cause of death (*i*) and the distribution of the endorsement rate for item *j* among all other causes in the analysis cause list:

$$ \frac{\begin{array}{c}\hfill Tarif{f}_{cause\kern0.5em i,\kern0.5em  item\kern0.5em j}=\hfill \\ {}\hfill Endorsement\kern0.5em  Rat{e}_{cause\kern0.5em i,\kern0.5em  item\kern0.5em j}- Median\kern0.5em  Endorsement\kern0.5em  Rat{e}_{item\kern0.5em j}\hfill \end{array}}{Interquartile\kern0.5em  Rang{e}_{item\kern0.5em j}} $$

To assign a cause to a death, we compute summed tariff scores for each cause in the analysis cause list based on the distribution of endorsed items for that death:

$$ Tariff\kern0.5em  Scor{e}_j^k={\displaystyle \sum_{r=1}^{40} Tarif{f}_{j\kern0.5em i}^{(r)}*{x}_{k\kern0.5em i}^{(r)}} $$

where *k* is the given decedent, *i* is the item, and *j* is the cause of death, *x*_*ki*_ is the response for decedent *k* on item *i*, with a value of 1 for a positive response and 0 for a negative response, and *r* identifies the specific item being used among top 40 with the highest absolute tariffs for cause *j*. Tariff scores for a given decedent are computed for every possible COD.

Therefore, the tariff score of an item for a given cause will depend on its endorsement rate, and some causes will have inherently high tariffs. For example, the item “Decedent suffered poisoning” has a strong association with a few causes of death (poisoning and suicide) and carries high tariffs for those causes. On the other hand, the item “Decedent had a rash” is associated with many different causes of death and carries low tariffs for the causes it is associated with.

A tariff score is calculated for all causes for a given decedent. The most obvious way to assign cause of death would be to select the one that carries the highest (summed) tariff score. However, some causes carry inherently higher tariffs than do others. Therefore to make the tariff scores for different causes comparable, all deaths in the training dataset were ranked by their tariff scores from highest to lowest, and the tariff score for a decedent was compared with these ranks. The cause with the highest ranked tariff score was assigned to the decedent; this makes use of all the information in the training dataset to normalize tariff scores.

The Tariff Method (both Tariff 1.0 and Tariff 2.0) is trained using the validated PHMRC database for which VAs were collected for deaths with hospital records fulfilling the gold standard criteria (validated VAs). During the development of both Tariff 1.0 and 2.0, however, the PHMRC gold standard dataset was repeatedly divided into a training dataset (from which methods were developed) and a testing dataset (used to test the performance of the methods).

### Tariff 2.0

Tariff 2.0 follows the same process as described above in assigning CODs, but improves on Tariff 1.0 in four important ways.

1. **Significance testing for each tariff**
  One limitation of Tariff 1.0 is that items that are strongly associated with a small number of deaths in the PHMRC database can drive COD assignments. To address this issue, we created 500 bootstrapped samples of the dataset with replacement of all symptoms by cause up to the original sample size. We then used the 500 samples to generate a 95 % uncertainty interval (UI) around each tariff estimate and removed tariffs with uncertainty intervals that included zero.
2. **Standardization of text mining**
  Standardization of text mining is an iterative process that involves making changes to data preparation and empirically testing how these changes affected model performance. For text analysis, all text were translated to English before starting data mining. We first identified key words that appeared at least 50 times within the open-ended narrative using the Text Mining package in R (version 2.14.0) [14]. Second, we grouped words to form items by stemming (e.g. “injuries” and “injured” formed an item, “injuri”) and also grouped words with similar meanings (e.g. “fire” and “burn”). We calculated tariffs for each of these text items. A physician then reviewed text items with statistically significant tariffs for clinical plausibility. These belonged, broadly, to three groups: obvious symptom items; items which appeared to be based on HCE; and other items, often with high tariffs, but with no obvious biological association. For example, the text item “road” had a tariff of 6.5 for road traffic accidents but also had a tariff of 3.0 or more for a number of cancers. The spurious association between “road” and “cancer” arose because of respondents mentioning the Ocean Road Cancer Institute in Dar es Salaam. Tariffs based on text items that were clinically implausible were removed from the analysis.
3. **Biologically and epidemiologically implausible cause assignments**
  We examined cause assignments at both individual and population levels. We disallowed biologically impossible cause assignments such as males with cervical cancer as well as highly unlikely assignments such as males with breast cancer. At the population level, we censored unlikely assignments such as malaria deaths in non-endemic regions. Additional file 2 lists the full set of exclusion criteria.
  We made very few changes to question items. We excluded a number of items, particularly those associated with health-seeking behavior, which had implausible associations with COD and were a consequence of the original dataset being hospital-based. For example, in the PHMRC validation dataset some gold standard deaths were obtained from police reports and coronial inquiries. However, when analyzing datasets from community deaths, an implausibly high percentage of population deaths had been attributed to drowning because decedents had not been taken to hospital.
4. **Indeterminate cause of death**
  Gold standard deaths were selected because they met predetermined criteria. It is probable that more information will be available about such cases than will be available for home deaths or, indeed, for other hospital deaths. An extreme example is of a 90-year-old woman whose relatives endorsed only a single question item: “Had her periods stopped naturally because of menopause?” Tariff 1.0 would assign causes that had few symptoms or had low average tariff scores to such a case. Because the assignment of drowning as the COD was driven by the single item: “Did the decedent suffer from drowning?”, the woman was initially assigned drowning as the COD. Overall, 29 of 40 items for drowning carried negative tariffs. Cases with little information, i.e., with multiple negative responses to question items, were thus attracted to drowning as a COD.
  To address this problem, using the training dataset, which was sampled with replacement to create a uniform cause distribution, we developed a method for identifying deaths where there was insufficient information from the VA interview to assign a COD and coded such deaths as indeterminate. At the ranking stage of analysis we stipulated that tariff scores for a given decedent needed to be above both cause-specific and absolute thresholds. If a tariff score was below either the cause-specific or the absolute threshold, that cause was disallowed for that decedent. If all causes were disallowed, the decedent was classified as indeterminate.
  We reallocated indeterminate deaths at the population level so that the sum of the CSMFs from all causes of death was 1.0. We did so based on 1) a Tariff model performance weight that was equal to the probability of a death from a given cause being assigned as indeterminate by the Tariff Method weighted by 2) a Global Burden of Disease (GBD) weight equal to the estimated distribution of cause-specific mortality by age and sex for a country in the GBD study 2010 [15]. This weight is used to calculate the fraction of an indeterminate death that is allocated to each COD. Weights sum to one. To illustrate this process, Tariff and GBD weights for a 45-year-old male in the Philippines are shown in Additional file 3. In this example, for cirrhosis the average of the GBD weight (0.054) and the tariff weight (0.026) is used to generate an overall weight for cirrhosis (0.039). If 45-year-old male decedent from the Philippines then 0.039 would be added to the number of cirrhosis deaths when generating the population-level cause of death distributions. The same would be done using the other weights for the other causes. Thus, in Tariff 2.0, an indeterminate VA is partially reallocated to multiple causes of death to create population-level cause of death estimates that are representative of the population from which they came. We did not reallocate indeterminate deaths at the individual level.

### Performance metrics

The performance of methods for assigning COD is a function of the true COD composition in a study population [16]. The PHMRC study developed methods to assess performance independently of COD composition and, at the same time, account for random chance effects on COD composition [16]. The 500 train-test data analysis datasets, each with a different COD composition, were generated by holding 75 % of the dataset as “training” data and 25 % as “test” data. Each test dataset was sampled with replacement using a Dirichlet distribution to provide a new CSMF composition. There was no correlation between the COD composition of the train set and the test set. Additional file 4 illustrates how the validation data have been used to generate each train-test pair. A detailed account of this procedure is given elsewhere [16].

We use two metrics to assess the performance of a method: median chance-corrected concordance (CCC) and cause-specific mortality fraction (CSMF) accuracy [16]. The first quantifies performance in correctly predicting COD for an individual and the second in predicting COD composition in populations. Analysis of the 500 test datasets results in a distribution from which we calculate the two metrics and their uncertainty intervals. Results are not biased by the particular cause composition of the dataset.

We assessed the performance of the Tariff Method in correctly assigning a COD to an individual VA using CCC. CCC adjusts sensitivity for chance so that a prediction without error would equal 1 and with random allocation would equal 0. CCC is calculated as:

$$ CC{C}_j=\frac{\left(\frac{T{P}_j}{T{P}_j+F{N}_j}\right)-\left(\frac{1}{N}\right)}{1-\left(\frac{1}{N}\right)} $$

where *TP*_*j*_ is true positives or number of decedents with gold standard cause *j* correctly assigned to cause *j*, *FN* is false negatives or the number of decedents incorrectly assigned to cause *j*, and *N* is the number of causes analyzed. *TP* plus *FN* equals the true number of deaths due to cause *j*.

Performance was also measured at the population level using the mean CSMF accuracy across the 500 cause compositions:

$$ CSMF\kern0.5em  Accuracy=1-\frac{{\displaystyle {\sum}_{j=1}^k\left|CSM{F}_j^{true}-CSM{F}_j^{pred}\right|}}{2\left(1- Minimum\left(CSM{F}_j^{true}\right)\right)} $$

where the numerator is the sum of the absolute error for all *k* causes between the true CSMF and the estimated CSMF and the denominator is the maximum possible error across all causes. A prediction without error would result in CSMF accuracy = 1, whereas a totally erroneous prediction would result in CSMF accuracy = 0. In a further development, we also estimated the CSMF accuracy, correcting by chance, namely chance-corrected CSMF (CCCSMF) accuracy [17].

## Results

### Validation of Tariff 2.0

Although the most important practical application of VAs lies in the prediction of the cause composition of mortality at the level of the population (CSMFs), the focus of this paper will be on an analysis of the effects of revisions to Tariff Method upon the different causes of death at the level of the individual person (median CCC). Such a detailed analysis is not possible at the population level.

Tables 3 and 4 provide an overview of results by CSMF accuracy and median CCC, respectively. For both metrics, and for each of three modules with and without HCE, Tariff 2.0 performs significantly better than did Tariff 1.0. Improvements were most notable in children and neonates but, also, statistically significant in adults. Thus, improvement in CSMF accuracy with HCE was 2.5 %, 7.4 %, and 14.9 % for adults, children, and neonates, respectively, and for median CCC with HCE it was 6.0 %, 13.5 %, and 21.2 %, respectively. Similar levels of improvement are seen in results with no HCE. Differences in improvement between CSMF accuracy and median CCC are more apparent than real. If CSMF accuracy in adults is corrected to take random allocation of COD into account, or CCCSMF accuracy with HCE, improvements are 6.8 %, 20.1 %, and 40.3 % for adults, children, and neonates, respectively.

**Table 3:** Median CSMF and CCCSMF accuracy across 500 splits

		Tariff 1.0				Tariff 2.0				Difference in CSMF accuracy (%)	Difference in CCSMF accuracy (%)
		Median CSMF accuracy (%)	95 % UI	Median CCCSMF accuracy (%)	95 % UI	Median CSMF accuracy (%)	95 % UI	Median CCCSMF accuracy (%)	95 % UI		
Adult	No HCE	69.5	(69.0, 69.9)	17.1	(15.8, 18.2)	71.7	(71.1, 72.1)	23.1	(21.6, 24.3)	2.2	6.0
	HCE	74.5	(73.9, 75.3)	30.7	(29.1, 32.9)	77.0	(76.6, 77.5)	37.6	(36.5, 38.9)	2.5	6.8
Child	No HCE	64.2	(63.5, 65.1)	2.7	(0.8, 5.2)	74.4	(73.6, 75.1)	30.5	(28.4, 32.4)	10.2	27.7
	HCE	70.9	(70.4, 71.5)	20.9	(19.6, 22.6)	78.3	(77.6, 78.7)	41.1	(39.2, 42.0)	7.4	20.1
Neonate^a^	No HCE	66.3	(65.5, 67.1)	8.4	(6.3, 10.6)	81.3	(80.7, 82.4)	49.2	(47.4, 52.2)	15.0	40.8
	HCE	67.9	(67.0, 68.9)	12.8	(10.3, 15.5)	82.8	(81.9, 83.5)	53.1	(50.9, 55.1)	14.9	40.3

^a^Tariff 1.0 has 11 causes for neonates vs. the six used for Tariff 2.0

**Table 4:** Median CCC across 500 splits

		Tariff 1.0		Tariff 2.0		
		Median (%)	95 % UI	Median (%)	95 % UI	Difference
Adult	No HCE	34.3	(34.1, 34.5)	37.8	(37.6, 37.9)	3.5
	HCE	44.5	(44.2, 44.7)	50.5	(50.2, 50.7)	6.0
Child	No HCE	28.8	(28.4, 29.2)	44.6	(44.2, 45.0)	15.8
	HCE	39.0	(38.4, 39.4)	52.5	(52.1, 53.0)	13.5
Neonate^a^	No HCE	21.6	(21.2, 22.2)	42.3	(41.9, 42.6)	20.7
	HCE	23.9	(23.6, 24.4)	45.1	(44.6, 45.4)	21.2

^a^Tariff 1.0 has 11 causes for neonates vs. the six used for Tariff 2.0

Median CCC for Tariff 1.0 and 2.0 with and without HCE is shown in Table 5 for adults and in Additional files 5 and 6 for children and neonates, respectively. It should be noted that the allocation of deaths to an indeterminate category will reduce median CCC but increase the accuracy of CSMFs in Tariff 2.0.

**Table 5:** Median CCC by cause of death: adults

Adult causes	No HCE		HCE		HCE difference
	Median (%)	95 % UI	Median (%)	95 % UI	
GBD cause group A: Communicable and maternal disorders					
Maternal	65.7	(64.9, 66.4)	68	(67.3, 68.4)	2.3
Malaria	29.9	(29.9, 29.9)	57.9	(55.2, 58.8)	28
AIDS	18.4	(17.8, 18.9)	51	(50.5, 51.8)	32.6
Tuberculosis	21	(20.5, 21.6)	43.5	(43.1, 44.3)	22.5
Diarrhea/dysentery	33.1	(32.1, 33.8)	38.5	(37.8, 39.3)	5.4
Other infectious diseases	3.2	(3.0, 3.3)	15.9	(15.5, 16.6)	12.7
Pneumonia	4.6	(4.0, 4.9)	15.2	(14.7, 15.5)	10.6
GBD cause group B: Non-communicable diseases					
Esophageal cancer	58.8	(58.8, 58.8)	79.4	(79.4, 79.4)	20.6
Cirrhosis	69.4	(68.3, 70.9)	75.8	(73.6, 76.2)	6.4
Breast cancer	70.6	(69.8, 70.9)	74.8	(74.8, 76.4)	4.2
Prostate cancer	48.5	(48.5, 48.5)	65.7	(62.5, 65.7)	17.2
Asthma	31.3	(31.3, 31.3)	57.1	(57.1, 65.7)	25.8
Epilepsy	39.9	(39.9, 48.5)	57.1	(57.1, 57.1)	17.2
Colorectal cancer	17.3	(16.7, 18.1)	51.2	(50.5, 51.9)	33.9
Diabetes	31	(30.3, 31.3)	50.9	(50.2, 51.6)	19.9
Stroke	43.3	(42.9, 43.8)	50.4	(49.8, 51.0)	7.1
Acute myocardial infarction	41.2	(40.4, 42.2)	44.4	(43.5, 44.9)	3.2
Cervical cancer	20.9	(19.6, 20.9)	40.1	(38.7, 40.1)	19.2
Other cardiovascular diseases	16.5	(15.9, 17.0)	37.3	(36.4, 38.0)	20.8
Leukemia/lymphomas	18.1	(17.6, 18.1)	34.9	(34.0, 36.6)	16.8
Stomach cancer	16.3	(16.3, 16.3)	29.2	(29.2, 35.6)	12.9
Renal failure	5	(4.7, 5.4)	28.9	(28.5, 29.6)	23.9
Lung cancer	12.8	(9.8, 12.8)	28.7	(28.7, 28.7)	15.9
Chronic obstructive pulmonary disease	5.2	(5.2, 5.2)	17.6	(17.6, 17.6)	12.4
Other non-communicable diseases	7	(6.7, 7.3)	14.6	(14.1, 15.0)	7.6
GBD cause group C: Injuries					
Road traffic	76.9	(76.0, 77.3)	81.5	(81.5, 82.5)	4.6
Drowning	84.1	(84.1, 84.1)	81.3	(80.2, 84.1)	−2.8
Bite of venomous animal	87.1	(87.1, 87.1)	80.7	(80.7, 80.7)	−6.4
Homicide	72.2	(70.6, 73.0)	78.4	(77.9, 79.9)	6.2
Other injuries	68.3	(64.3, 68.3)	72.3	(69.1, 72.3)	4
Fires	72.5	(72.5, 72.5)	71.7	(69.1, 72.5)	−0.8
Falls	58.3	(56.9, 59.3)	59.3	(58.8, 60.4)	1
Poisonings	34.4	(31.3, 34.4)	57.9	(55.8, 57.9)	23.5
Suicide	7.1	(6.9, 8.4)	9.8	(7.6, 10.3)	2.7
Summary					
Group A	25.1	(24.9, 25.5)	41.3	(41.0, 41.6)	16.2
Group B	30.8	(30.4, 31.0)	46.7	(46.5, 47.0)	15.9
Group C	61.9	(61.8, 62.5)	65.3	(65.1, 65.8)	3.4
Total	37.8	(37.6, 37.9)	50.5	(50.2, 50.7)	12.7

We describe here results for adult causes of death in detail. In general, median CCC is higher in children and neonates because fewer causes of death are reported. Table 5 shows median CCC for Tariff 2.0 with and without HCE for 34 adult causes grouped according to broad GBD cause categories: A. Communicable, maternal, neonatal, and nutritional disorders; B. Non-communicable diseases; and C. Injuries. Causes have been ranked by median CCC with HCE for Tariff 2.0 within categories.

Group C has higher median CCC with HCE (65.3 %) than do Groups B (46.7 %) and A (41.3 %). On average, HCE made an important contribution to median CCC for Group A (16.2 %) and Group B (15.9 %), but not to Group C (3.4 %).

Group A contained only six specific causes and a residual group. All these specific causes are associated with global programs for their control.

1. The diagnosis of maternal death was least dependent on HCE being correctly assigned in 68.0 % of cases with HCE and 65.7 % of cases without HCE. The model was, however, unable to distinguish between different causes of maternal death: obstructed labor; hemorrhage; sepsis; and anemia.
2. Median CCC was mid-range for malaria (57.9 %), AIDS (51.0 %), and pulmonary tuberculosis (43.5 %). Malaria and AIDS are difficult to characterize for purposes of VA because they can affect many different organs and can present with any one of a number of different syndromes. On the other hand, it is difficult to distinguish clinically between pulmonary tuberculosis and other chronic lung diseases because of their common effects on the lungs. It was not surprising that correct diagnosis for these three diseases depended heavily on HCE. Median CCC increased with HCE by 28.0 % for malaria, 32.6 % for AIDS, and 22.5 % for pulmonary tuberculosis. Text and question items about fever attracted low tariffs across a range of infectious causes. Probably in consequence, 9.3 % of gold standard deaths from malaria were classified as indeterminate by Tariff 2.0.
3. Diarrheal diseases and pneumonia have well-defined clinical presentations, yet median CCC with HCE was low-range: diarrhea/dysentery (38.5 %) and pneumonia (15.2 %). This appeared to be a consequence of many different respiratory symptoms attracting low tariffs because of their wide distribution among different causes. The model was unable to distinguish between diarrhea and dysentery.
4. The residual category, “other infectious diseases”, also performed poorly (15.9 % with HCE, 3.2 % without).

Group B contained eight cancers, eight other non-communicable diseases, and two residual categories. Nearly all are chronic conditions that are not stigmatized, and families are likely to know and be open about the diagnosis.

1. Median CCC was greatest for three cancers: esophagus (79.4 %); breast (74.8 %); and prostate (65.7 %). It was mid-range for colorectal cancer (51.2 %) and cervical cancer (40.1 %) and low-range for leukemia and lymphomas (34.9 %), stomach cancer (29.2 %), and lung cancer (28.7 %). The effect of HCE was greatest for colorectal cancer (33.9 %) and least for breast cancer (4.2 %); the effect of HCE was in the range 12.9–20.6 % for the remaining cancers.
2. Median CCC was high for cirrhosis (75.8 %): this high score was associated with jaundice, alcoholism, bleeding from esophageal varices, and a protruding abdomen (ascites); HCE made only a 3.8 % contribution. Median CCC with HCE was mid-range for five diseases: asthma (57.1 %); epilepsy (57.1 %); diabetes (50.9 %); stroke (50.4 %); and acute myocardial infarction (44.4 %). HCE contributed 25.8 % to asthma diagnosis, 17.2 % to epilepsy, 19.9 % to diabetes, but only 7.1 % to stroke. Median CCC for renal failure was 28.9 % with HCE and 5.0 % without HCE; for chronic obstructive pulmonary disease (COPD) it was 17.6 % with HCE and 5.2 % without.
3. Median CCC for “other cardiovascular diseases” was 37.3 % and for “other non-communicable diseases”, 14.6 %. This latter residual category includes cancers because the model was unable to distinguish between other cancers and other non-communicable diseases.

Group C contained external causes of death. Six causes were due to accidents and two (homicide and suicide) to intentional acts.

1. Median CCC was high for road traffic accidents (81.5 %), drowning (81.3 %), bite of venomous animal (80.7 %), homicide (78.4 %), and fires (71.7 %).
2. Median CCC was mid-range for falls (59.3 %) and poisonings (57.9 %). The HCE effect for poisonings was 28.5 %.
3. Median CCC was only 9.8 % for suicide, possibly reflecting stigmatization both with the gold standard cases and with the VA.

Tables 6 and 7 provide more information about endorsement rates and tariffs for gold standard maternal deaths. Table 6 shows endorsement rates for five key questions that define maternal death; in 20.1 % of cases respondents gave a negative response to all five. Specificity for maternal death was 99.3 %. Table 7 shows how tariffs distinguish maternal causes from cervical cancer but do not discriminate among maternal causes.

**Table 6:** Endorsement rates for question items that define a maternal death: gold standard maternal deaths

Question	Endorsement rate
Was [name] pregnant at the time of death?	27.1 %
Did [name] die during an abortion?	2.5 %
Did she die during labor or delivery?	10.4 %
Did she die within 6 weeks after having an abortion?	7.6 %
Did she die within 6 weeks of childbirth?	49.0 %
Answered “no” to these five maternal questions	20.1 %

**Table 7:** Comparison of tariffs for five different causes of maternal death with tariffs for cervical cancer

Symptom or text item	Anemia	Hemorrhage	Hypertensive disorder	Other maternal causes	Sepsis	Cervical cancer
Death during labor or delivery	191	52.5	141.5	121.5	0	0
Excessive bleeding after delivery or abortion	83	145.5	50	36.5	59.5	0
Duration of labor	132.5	0	53	75	0	0
Excessive bleeding during labor or delivery	73.5	61.5	62	46.5	0	0
Duration of pregnancy	77	34	68.5	51.5	0	0
Death within 6 weeks of childbirth	57.5	67.5	66	39	72	0
Bleeding during pregnancy	0	52.5	62	84	0	0
word_cesarean	0	52.5	71	65.5	0	0
Pregnant at the time of death	36.5	17	34.5	35.5	14.5	0
word_pregnanc	27	21.5	27	36	23.5	0
word_deliv	24	35	18.5	25.5	26.5	0
Death within 6 weeks of an abortion	30.5	25.5	0	39	64.5	0
word_babi	0	20	15	27	0	0
Excessive vaginal bleeding in week before death	11	23.5	8	13	9	5.5
Death during an abortion	0	44	0	0	0	0
word_womb	17	0	10.5	14	19	0
How many weeks was her period overdue?	8	13	9	9.5	6	0
word_birth	0	14	4	11	12.5	0
Vaginal bleeding other than her period	4.5	11.5	3.5	7.5	6	7

### Community VAs

All population VA data was analyzed by site and module. The age and sex distribution of decedents in the community dataset was comparable to that of the gold standard dataset, although the adults and neonates were slightly older. The percentage of decedents who sought care outside of the home was lower for all modules (see Additional file 7).

## Discussion

It is essential to recognize that Tariff Method has been formally validated against the PHMRC gold standard database. Through validation it has been possible to compare accuracy between different analytic methods for assigning COD and, in this paper, to assess in detail the effect of revisions to Tariff Method. We have demonstrated increased accuracy of Tariff Method for CSMFs of 2.2 % and 2.5 % for adult modules with and without HCE, of 10.2 % and 7.4 % for child modules, and of 15.0 % and 14.9 % for neonatal modules. We have also shown increased accuracy for median CCC of 3.5 % and 6.0 % for adult modules with and without HCE and of 20.7 % and 21.2 % for neonatal modules.

Random allocation of deaths to different causes would result in CSMF accuracy of 0.632. These results were obtained by randomly assigning CODs from the reporting cause lists to 500 simulated populations with different cause compositions. If CSMF accuracy with HCE shown in Table 3 is adjusted to show improvement over random chance (CCCSMF accuracy), then adjusted accuracy would be 37.6 %, 41.1 %, and 53.1 % for adults, children, and neonates, respectively. To put these results into perspective, the reported CSMF accuracy of medical certification of adult deaths in Mexican teaching hospitals was 82 % [18]; this is equivalent to an adjusted accuracy of 50 %.

Creation of the validation dataset has also made it possible to make objective judgments about the capacity of Tariff Method to discriminate between different CODs. The outcome has been the reporting cause list (Additional file 1). It has also been possible to identify those question and text items that contribute significantly to cause assignment and those that do not. We will be presenting details of a validated item-reduced instrument in a future communication.

In a recent article, Byass drew attention to some of the shortcomings of the PHMRC gold standard database [11]. He argued that although the internal validity of the dataset has been demonstrated, its external validity is suspect and in consequence there has been “over-fitting” of the empirical methods to the dataset. This argument was based in part on an earlier publication that pointed to the effects of small sample size (796 cases) on external validity [19]. There are no absolute criteria for external validity. The PHMRC dataset contained over 12,500 cases from four different countries. The first step in establishing external validity was the “out-of-sample” analyses involving the development of 500 datasets with stochastically determined distribution of causes. The second step was taken with the research described in this paper in which the revision of Tariff Method was, in the main, based on two sets of community VAs, but the validation was dependent on the original PHMRC database. A third step will be to add new gold standard hospital deaths to the PHMRC database.

Cases in the gold standard database serve to establish defining characteristics of a disease for the subsequent assigning of COD from verbal autopsies. To an extent they are the equivalent of type specimens in biology. Signs and symptoms featured prominently in the diagnostic criteria for many of the target diseases. Cases were selected because they met predetermined criteria; many cases were rejected. A key issue in developing Tariff 2.0 was to establish the minimum set of information that would define death by a particular cause. Although endorsement rates in the community VA datasets were comparable with the gold standard validation data, endorsement rates for some individual community deaths were very low. This led to the development of ranking cutoffs which allowed us to identify deaths where there was too little information for diagnosis and classify these as “indeterminate”.

Maternal deaths are a case in point. In 20 % of verbal autopsies for gold standard maternal death in the PHMRC database there was no response to any of five key questions that depend on knowledge of the pregnancy status of the decedent and serve to define a maternal death (Table 6). Byass has suggested that empirical methods can “learn” wrong conclusions: in this case, that non-pregnant women can die from maternal causes [11]. High specificity for maternal deaths indicates that this did not happen. Tariff-assigned CODs are the result of an additive process. The problem appears to be one of paucity of information in many verbal autopsies, possibly due to respondents’ lack of familiarity with the symptoms of the terminal illness or, in the case of maternal deaths, that the decedent was in fact pregnant. Such problems are more likely to arise in civil registration systems than in longitudinal population studies where the fact and outcome of pregnancy can be determined by other means. However, a comparison of tariffs between maternal causes and cervical cancer shows little room for confusion in assigning COD (Table 7): the problem for Tariff Method was that VA symptoms were distributed among a range of maternal causes and Tariff was unable to distinguish among them. The use of gold standards makes this problem explicit.

Byass also raises the question of whether hospital deaths provide a valid basis for the development of empirical methods to assign COD to verbal autopsies taken from open populations [11]. The present study was a response to just this situation. We have argued above that the principal problem was one of paucity of information and that Tariff 2.0 copes well under these circumstances. However, two other factors may come into play. The first is that the characteristics of a terminal illness, in particular its duration, may be altered through hospitalization. This would be truer for acute illnesses of childhood when disease characteristics do not have time to develop than for chronic illnesses where respondents would have had time to have become familiar with long-standing symptoms of the underlying cause of death. We can only point out that in children median CCC for infections (mostly acute) showed about 10 % increase between Tariff 1.0 and Tariff 2.0, and that the gold standards were heavily influenced by Integrated Management of Childhood Illness criteria. Certainly, CCC for deaths from respiratory causes in both adults and children was less than hoped. Byass attributes this to procedures during hospitalization resulting in generally high endorsement rates for respiratory symptoms. As likely are the generally high endorsement rates for hospital deaths referred to above. Another factor associated with hospitalization is that with certain disorders families lose contact with the decedent during their terminal illness. This would be particularly true for maternal and neonatal deaths.

In our view the benefits of a formal validation process based on gold standard cases far outweigh any disadvantages. No longer should agreement between analytic methods be regarded as a form of validation. As it has been discussed before, we consider CCC a more appropriate metric than Kappa to assess the performance of a VA instrument at the individual level [16]. Because performance metrics are dependent on disease prevalence it is essential that metrics be based on multiple comparisons produced through “splits” and not on a single comparison.

We foresee a number of areas where Tariff Method can continue to be improved. They include improvements to the analysis of open text, better analysis of the duration of illness, and expansion of the gold standard database. A final step in ensuring external validity would be to establish gold standards for deaths under conditions where family members would be likely to be present, e.g., in health centers.

The Institute for Health Metrics and Evaluation has made publicly available an electronic version of the PHMRC survey based on the Open Data Kit (ODK) platform [20]. Tariff 2.0 can be applied to the output of this mobile application, and population estimates can be generated through the SmartVA application in a matter of minutes. This is a vast improvement on the months or years that are often required for physician review, which has led to serious delays in the availability of data about COD in populations without reliable vital registration systems. Both the electronic survey and the SmartVA application can be found at this website: http://www.healthdata.org/verbal-autopsy/tools.

## Conclusions

Tariff 2.0 addresses the main shortcomings of the application of the Tariff Method to analyze data from VAs in community settings. Tariff 2.0 provides an estimation of COD from VAs with better performance at the individual and population level than the previous version of this method, and it is publicly available for use.

## Additional files

Additional file 1:
**List of causes of death of PHMRC.** (DOCX 16 kb) Additional file 2:
**Cause of death exclusion criteria for Tariff 2.0.** (DOCX 13 kb) Additional file 3:
**Tariff and GBD weights for a** **45-year-old** **male in the Philippines.** (DOCX 15 kb) Additional file 4:
**Flowchart for construction of** **train-test** **dataset from validation dataset.** (DOCX 120 kb) Additional file 5:
**Chance-corrected concordance for 21 child causes.** (DOCX 19 kb) Additional file 6:
**Chance-corrected concordance for 11 neonate causes.** (DOCX 16 kb) Additional file 7:
**Descriptive information comparing community VAs to gold standard validation data.** (DOCX 17 kb)

## Abbreviations

CCC: chance-corrected concordance
CCCSMF: chance-corrected cause-specific mortality fraction
COD: cause of death
CSMF: cause-specific mortality fraction
GBD: Global Burden of Disease
HCE: health care experience
ICD: International Classification of Diseases
NHMRC: National Health and Medical Research Council
ODK: Open Data Kit
PCVA: physician-certified verbal autopsy
PHMRC: Population Health Metrics Research Consortium
UI: uncertainty interval
VA: verbal autopsy
VAI: verbal autopsy instrument
