# Supplementary material for: Improving performance of the Tariff Method for assigning causes of death to verbal autopsies
Source: BMC Med. 2015 Dec 8;13:291. doi: 10.1186/s12916-015-0527-9 (PMC4672473; doi:10.1186/s12916-015-0527-9)
Supplement: Additional file 1: — List of causes of death of PHMRC. (DOCX 16 kb) [file 12916_2015_527_MOESM1_ESM.docx]

Additional file 1

List of causes of death for the Population Health Metrics Research Consortium including the 11-cause and 6-cause lists for neonates.

| **Adult Cause List** | **Child Cause List** | **Neonate cause lists** | |
| --- | --- | --- | --- |
|  |  | **11 causes** | **6 causes** |
| AIDS | AIDS | Birth asphyxia | Birth asphyxia |
| Asthma | Bite of Venomous Animal | Congenital malformation | Congenital malformation |
| Bite of Venomous Animal | Diarrhea/Dysentery | Meningitis/Sepsis | Meningitis/Sepsis |
| Breast Cancer | Drowning | Sepsis with Local Bacterial Infection | Meningitis/Sepsis |
| Cervical Cancer | Encephalitis | Pneumonia | Pneumonia |
| Cirrhosis | Falls | Preterm Delivery (without RDS) and Birth Asphyxia | Birth asphyxia |
| Colorectal Cancer | Fires | Preterm Delivery (with or without RDS) and Sepsis | Meningitis/Sepsis |
| COPD | Hemorrhagic fever | Preterm Delivery (without RDS) and Sepsis and Birth Asphyxia | **(DROPPED)** |
| Diabetes | Malaria | Preterm Delivery with Respiratory Distress Syndrome | Preterm Delivery |
| Diarrhea/Dysentery | Measles | Preterm Delivery without Respiratory Distress Syndrome | Preterm Delivery |
| Drowning | Meningitis | Stillbirth | Stillbirth |
| Epilepsy | Other Cancers |  |  |
| Esophageal Cancer | Other Cardiovascular Diseases |  |  |
| Falls | Other Defined Causes of Child Deaths |  |  |
| Fires | Other Digestive Diseases |  |  |
| Homicide | Other Infectious Diseases |  |  |
| Acute Myocardial Infarction | Pneumonia |  |  |
| Leukemia/Lymphomas | Poisonings |  |  |
| Lung Cancer | Road Traffic |  |  |
| Malaria | Sepsis |  |  |
| Maternal | Violent Death |  |  |
| Other Cardiovascular Diseases |  |  |  |
| Other Infectious Diseases |  |  |  |
| Other Injuries |  |  |  |
| Other Non-communicable Diseases |  |  |  |
| Pneumonia |  |  |  |
| Poisonings |  |  |  |
| Prostate Cancer |  |  |  |
| Renal Failure |  |  |  |
| Road Traffic |  |  |  |
| Stomach Cancer |  |  |  |
| Stroke |  |  |  |
| Suicide |  |  |  |
| TB |  |  |  |
