# Supplementary material for: Improving performance of the Tariff Method for assigning causes of death to verbal autopsies
Source: BMC Med. 2015 Dec 8;13:291. doi: 10.1186/s12916-015-0527-9 (PMC4672473; doi:10.1186/s12916-015-0527-9)
Supplement: Additional file 2: — Cause of death exclusion criteria for Tariff 2.0. (DOCX 13 kb) [file 12916_2015_527_MOESM2_ESM.docx]

Additional file 2

Cause of Death Exclusion Criteria for Tariff 2.0

- Breast cancer in males
- Maternal causes of death in males
- Prostate cancer in females
- Stillbirth deaths over the age of 0 days
- Malaria in non-endemic regions.
  - Within the GS data set, malaria was disallowed for Bohol, Philippines and Mexico City, Mexico
- Measles in Mexico
