# Supplementary material for: Improving performance of the Tariff Method for assigning causes of death to verbal autopsies
Source: BMC Med. 2015 Dec 8;13:291. doi: 10.1186/s12916-015-0527-9 (PMC4672473; doi:10.1186/s12916-015-0527-9)
Supplement: Additional file 3: — Tariff and GBD weights for a 45-year-old male in the Philippines. (DOCX 15 kb) [file 12916_2015_527_MOESM3_ESM.docx]

Additional file 3

Tariff and GBD weights for a 45 year old male in the Philippines

| Adult Cause | Tariff weight | GBD weight | Overall Weight |
| --- | --- | --- | --- |
| AIDS | 0.028 | 0.001 | 0.0012 |
| Asthma | 0.020 | 0.011 | 0.0062 |
| Bite of Venomous Animal | 0.000 | 0.001 | 0.0000 |
| Cirrhosis | 0.026 | 0.054 | 0.0390 |
| Colorectal Cancer | 0.038 | 0.010 | 0.0103 |
| COPD | 0.052 | 0.012 | 0.0177 |
| Diabetes | 0.029 | 0.047 | 0.0384 |
| Diarrhea/Dysentery | 0.043 | 0.006 | 0.0076 |
| Drowning | 0.018 | 0.007 | 0.0037 |
| Epilepsy | 0.000 | 0.003 | 0.0000 |
| Esophageal Cancer | 0.022 | 0.002 | 0.0013 |
| Falls | 0.023 | 0.010 | 0.0065 |
| Fires | 0.007 | 0.002 | 0.0003 |
| Homicide | 0.017 | 0.081 | 0.0383 |
| IHD – Acute Myocardial Infarction | 0.020 | 0.169 | 0.0935 |
| Leukemia/Lymphomas | 0.044 | 0.009 | 0.0107 |
| Lung Cancer | 0.018 | 0.027 | 0.0134 |
| Malaria | 0.092 | 0.001 | 0.0020 |
| Maternal | 0.025 | 0.000 | 0.0000 |
| Other Cardiovascular Diseases | 0.045 | 0.055 | 0.0701 |
| Other Infectious Diseases | 0.038 | 0.022 | 0.0230 |
| Other Injuries | 0.030 | 0.012 | 0.0103 |
| Other Non-communicable Diseases | 0.078 | 0.154 | 0.3349 |
| Pneumonia | 0.054 | 0.036 | 0.0550 |
| Poisonings | 0.057 | 0.000 | 0.0006 |
| Prostate Cancer | 0.016 | 0.001 | 0.0003 |
| Renal Failure | 0.044 | 0.027 | 0.0336 |
| Road Traffic | 0.007 | 0.025 | 0.0047 |
| Stomach Cancer | 0.009 | 0.005 | 0.0013 |
| Stroke | 0.028 | 0.112 | 0.0881 |
| Suicide | 0.000 | 0.010 | 0.0001 |
| TB | 0.036 | 0.087 | 0.0880 |
