# Supplementary material for: Improving performance of the Tariff Method for assigning causes of death to verbal autopsies
Source: BMC Med. 2015 Dec 8;13:291. doi: 10.1186/s12916-015-0527-9 (PMC4672473; doi:10.1186/s12916-015-0527-9)
Supplement: Additional file 4: — Flowchart for construction of train-test dataset from validation dataset. (DOCX 120 kb) [file 12916_2015_527_MOESM4_ESM.docx]

Additional file 4. Flowchart for construction of train-test dataset from validation dataset.
