# Supplementary material for: Improving performance of the Tariff Method for assigning causes of death to verbal autopsies
Source: BMC Med. 2015 Dec 8;13:291. doi: 10.1186/s12916-015-0527-9 (PMC4672473; doi:10.1186/s12916-015-0527-9)
Supplement: Additional file 5: — Chance-corrected concordance for 21 child causes. (DOCX 19 kb) [file 12916_2015_527_MOESM5_ESM.docx]

Additional file 5: Chance-corrected concordance for 21 child causes

| **Child Causes** | **Tariff 1.0** | | | | **Tariff 2.0** | | | |
| --- | --- | --- | --- | --- | --- | --- | --- | --- |
|  | **No HCE** | | **HCE** | | **No HCE** | | **HCE** | |
|  | **Median** | **95% UI** | **Median** | **95% UI** | **Median** | **95% UI** | **Median** | **95% UI** |
| **GBD Cause Group A: Communicable, maternal, neonatal and nutritional disorders** | | | | | | | | |
| AIDS | 14.6 | (13.3, 15.8) | 55.1 | (52.8, 57.0) | 37 | (37.0, 37.0) | 58 | (58.0, 58.0) |
| Diarrhea/Dysentery | 35.4 | (34.0, 36.5) | 41.2 | (40.0, 42.7) | 24.6 | (24.0, 25.5) | 37 | (36.3, 38.2) |
| Encephalitis | 31.1 | (29.3, 33.1) | 31.1 | (29.3, 33.6) | 37 | (37.0, 37.0) | 41.7 | (37.0, 47.5) |
| Hemorrhagic fever | 43.9 | (41.8, 46.2) | 40.8 | (38.8, 42.8) | 51.5 | (51.5, 52.3) | 59.6 | (59.6, 65.0) |
| Malaria | 53.1 | (51.0, 55.2) | 61.8 | (60.0, 63.9) | 41.7 | (40.0, 42.1) | 57.2 | (56.6, 58.8) |
| Measles | 74.7 | (72.2, 76.9) | 74 | (70.0, 76.3) | 82.5 | (82.5, 82.5) | 82.5 | (82.5, 82.5) |
| Meningitis | 20.3 | (18.6, 21.7) | 24.5 | (22.9, 26.5) | 27.3 | (25.0, 32.5) | 30 | (26.5, 32.5) |
| Other Infectious Diseases | 1.4 | (0.7, 2.2) | 3.3 | (1.7, 4.9) | 7.4 | (7.4, 10.0) | 26.5 | (25.9, 27.8) |
| Pneumonia | 15.1 | (14.1, 16.0) | 24.6 | (23.4, 26.5) | 7.6 | (7.2, 8.1) | 9.9 | (9.1, 10.8) |
| Sepsis | 8.5 | (7.5, 10.0) | 7.4 | (6.7, 8.5) | 13.1 | (11.6, 13.5) | 14.7 | (14.1, 16.0) |
| **GBD Cause Group B: Non-communicable diseases** | | | | | | | | |
| Other Cancers | 4.1 | (-5.0, 6.3) | 24.7 | (23.0, 26.4) | 25 | (25.0, 30.0) | 40 | (40.0, 40.0) |
| Other Cardiovascular Diseases | 6.9 | (5.6, 8.2) | 26.2 | (24.5, 27.7) | 12.5 | (11.6, 13.5) | 33.7 | (33.7, 33.7) |
| Other Defined Causes of Child Deaths | 7.5 | (6.7, 8.6) | 15.9 | (15.3, 16.6) | 5.9 | (5.0, 6.4) | 16.9 | (16.0, 18.3) |
| Other Digestive Diseases | 13 | (11.9, 14.8) | 20.5 | (18.8, 21.4) | 3.8 | (3.8, 3.8) | 21.3 | (21.3, 21.3) |
| **GBD Cause Group C: Injuries** | | | | | | | | |
| Bite of Venomous Animal | 38.8 | (35.5, 40.0) | 56.3 | (53.1, 59.8) | 92.5 | (92.5, 92.5) | 92.5 | (92.5, 100.0) |
| Drowning | 40.2 | (38.6, 42.1) | 50.7 | (49.2, 52.2) | 90 | (89.5, 90.0) | 93.8 | (92.5, 94.7) |
| Falls | 58.4 | (55.9, 60.2) | 67 | (65.0, 69.5) | 73.8 | (73.8, 73.8) | 73.8 | (73.8, 73.8) |
| Fires | 47.5 | (44.3, 50.8) | 63.3 | (60.6, 65.8) | 69.1 | (69.1, 69.1) | 75.3 | (73.8, 75.3) |
| Poisonings | 22.1 | (20.8, 23.4) | 22.3 | (21.3, 23.7) | 47.5 | (47.5, 47.5) | 73.8 | (73.8, 73.8) |
| Road Traffic | 19.7 | (18.3, 21.5) | 53.3 | (51.0, 55.5) | 90.5 | (88.9, 90.9) | 90.9 | (90.9, 92.5) |
| Violent Death | 28.4 | (26.9, 30.5) | 52.6 | (49.6, 55.0) | 83.8 | (82.5, 83.8) | 83.8 | (83.8, 83.8) |
| **Summary** | | | | | | | | |
| Group A | 29.8 |  | 36.4 |  | 33.5 | (33.0, 34.1) | 42.2 | (41.6, 42.8) |
| Group B | 7.9 |  | 21.8 |  | 14 | (12.6, 14.3) | 28.3 | (27.9, 29.3) |
| Group C | 36.4 |  | 52.2 |  | 78.1 | (77.7, 79.0) | 81.1 | (81.1, 82.6) |
| Total | 28.8 | (28.4, 29.2) | 39 | (38.4, 39.4) | 44.6 | (44.2, 45.0) | 52.5 | (52.1, 53.0) |
