# Supplementary material for: Improving performance of the Tariff Method for assigning causes of death to verbal autopsies
Source: BMC Med. 2015 Dec 8;13:291. doi: 10.1186/s12916-015-0527-9 (PMC4672473; doi:10.1186/s12916-015-0527-9)
Supplement: Additional file 6: — Chance-corrected concordance for 11 neonate causes. (DOCX 16 kb) [file 12916_2015_527_MOESM6_ESM.docx]

Additional file 6: Chance-corrected concordance for 11 neonate causes

| **Neonate Causes** | **Tariff 1.0** | | | | **Tariff 2.0** | | | |
| --- | --- | --- | --- | --- | --- | --- | --- | --- |
|  | **No HCE** | | **HCE** | | **No HCE** | | **HCE** | |
|  | **Median** | **95% UI** | **Median** | **95% UI** | **Median** | **95% UI** | **Median** | **95% UI** |
| Birth asphyxia* | 22.5 | (21.2, 23.6) | 22.8 | (21.7, 23.6) | 22.1 | (21.5, 22.8) | 25.9 | (25.1, 26.6) |
| Congenital malformation | 30.4 | (28.8, 31.7) | 33.2 | (32.0, 34.5) | 34.1 | (32.3, 34.2) | 34.8 | (34.2, 36.1) |
| Meningitis/Sepsis* | 21.9 | (20.0, 23.5) | 20.3 | (19.0, 21.7) | 38.0 | (37.3, 39.0) | 44.3 | (43.2, 45.1) |
| Pneumonia | 12.0 | (10.7, 13.6) | 16.6 | (15.6, 18.1) | 31.4 | (31.4, 37.1) | 37.1 | (37.1, 37.1) |
| Preterm Delivery* |  |  |  |  | 41.7 | (41.0, 42.3) | 40.5 | (40.0, 41.1) |
| Preterm Delivery and Sepsis* | 19.2 | (17.0, 20.8) | 22.9 | (21.0, 24.7) |  |  |  |  |
| Preterm Delivery and Birth Asphyxia* | 11.4 | (10.2, 13.3) | 14.3 | (13.1, 15.8) |  |  |  |  |
| Preterm Delivery and Sepsis/Birth Asphyxia** | 41.5 | (37.6, 43.5) | 46.8 | (45.0, 51.1) |  |  |  |  |
| Preterm Delivery with RDS* | 28.0 | (26.1, 30.0) | 22.2 | (20.6, 23.9) |  |  |  |  |
| Preterm Delivery without RDS* | 8.0 | (6.9, 9.0) | 8.9 | (7.7, 10.2) |  |  |  |  |
| Stillbirth | 29.3 | (27.8, 31.0) | 28.7 | (27.0, 30.5) | 85.7 | (85.2, 86.2) | 85.6 | (85.1, 86.1) |

*Deaths due to preterm delivery with comorbidities were reported as five separate causes for Tariff 1.0. These were collapsed into their separate comorbidities for Tariff 2.0. Preterm delivery incorporated Preterm Delivery with RDS and Preterm Delivery without RDS.

** Preterm Delivery and Sepsis/Birth Asphyxia cases were dropped (34 cases total)
