# Supplementary material for: Improving performance of the Tariff Method for assigning causes of death to verbal autopsies
Source: BMC Med. 2015 Dec 8;13:291. doi: 10.1186/s12916-015-0527-9 (PMC4672473; doi:10.1186/s12916-015-0527-9)
Supplement: Additional file 7: — Descriptive information comparing community VAs to gold standard validation data. (DOCX 17 kb) [file 12916_2015_527_MOESM7_ESM.docx]

Additional file 7: Descriptive information comparing community VAs to GS Validation data

| **Module** | **Dataset** | **Average Age** | **Percent who sought care outside the home** | **Percent female** | **Sample size** | **TABLE 2 (n)** |
| --- | --- | --- | --- | --- | --- | --- |
| **Adult** | GS Validation Data | 58.7 years | 94.2% | 45.6% | 7846 | 2680 |
| **Child** | GS Validation Data | 2.7 years | 89.8% | 44.9% | 2064 | 222 |
| **Neonate** | GS Validation Data | 2.8 days | 62.2% | 43.9% | 2625 | 165 |
| **Adult** | Community VAs | 61.0 years | 73.8% | 43.7% | 10987 | 8307 |
| **Child** | Community VAs | 2.9 years | 80.2% | 45.6% | 769 | 547 |
| **Neonate** | Community VAs | 13.4 days | 55.9% | 42.8% | 772 | 607 |
|  | | | |  | 25063 | 12528 |
